# Monitoring of Mycotoxigenic Fungi in Fish Farm Water and Fumonisins in Feeds for Farmed *Colossoma macropomum*

**DOI:** 10.3390/toxics11090762

**Published:** 2023-09-08

**Authors:** Juliana Sousa Terada-Nascimento, Jerônimo Vieira Dantas-Filho, Bruna Lucieny Temponi-Santos, Vinícius Perez-Pedroti, Maria Mirtes de Lima Pinheiro, Ricardo Ysaac García-Nuñez, Igor Mansur Muniz, Átila Bezerra de Mira, Elica Amara Cecilia Guedes, Sandro de Vargas Schons

**Affiliations:** 1 Programa de Pós-Graduação em Ciências Ambientais, Universidade Federal de Rondônia, UNIR, Rolim de Moura 76.940-000, Brazil; julianaterada1@gmail.com (J.S.T.-N.); sandroschons@unir.br (S.d.V.S.); 2 Grupo de Pesquisa em Patologia Animal no Bioma Amazônico, Centro de Diagnóstico Animal, Universidade Federal de Rondônia, UNIR, Rolim de Moura 76.940-000, Brazil; 3 Departamento de Medicina Veterinária—Zootecnia, Universidad Nacional Amazónica de Madre de Dios, UNAMAD, Puerto Maldonado 17.0001-000, Peru; 4 Centro de Ciências Agrárias e Instituto de Ciências Biológicas e da Saúde, Universidade Federal de Alagoas, UFAL, Maceió 57.480-000, Brazil

**Keywords:** aquatic microbiology, ecotoxicology, fish feed, mycotoxins

## Abstract

This study aimed to evaluate the occurrence of mycotoxigenic fungi in fish farm water and mycotoxins in feeds for farmed tambaqui (*Colossoma macropomum*). A total of 40 samples of freshwater from fish farms and 16 samples of feed were collected and analyzed for microbiology. A total of five species of free-living fungi were identified in fish farms: *Aspergillus fumigatus*, *Penicillium citrinum*, *P. implicatum*, *Fusarium oxysporum* and *Alternaria alternata*. These fungi species were counted in water samples at 35.14 CFU mL^−1^ and 24.69 CFU mL^−1^ in the dry seasons. In all fish farms, there was a higher abundance of fungi species in the rainy season. During visits to the fish farmers, it was possible to verify poor feed storage conditions. Concerning mutations in blood cells, in tambaqui (*C. macropomum*), a total of 159 anomalies were found, and in *Leptodactylus petersii,* 299 anomalies were found, with higher incidences in conditions above 1.0 CFU mL^−1^ in log_10_^(x+1)^ fungi and in the rainy season. The occurrence of mycotoxicological contamination was confirmed in 81.25% of the analyzed samples. The quantified mycotoxin was Fumonisins B_1_ + B_2_ (375 to 1418 μg kg^−1^). Pearson’s correlation analysis showed a significant positive correlation between Fumonisins and feed samples (r = 0.83). There was also a significant positive correlation between the abundance of fungi in water and the quantification of Fumonisins (r = 0.79). Based on the results obtained, it can be concluded that free-living fungi can be used as bioindicators of water quality in fish farms. Consequently, the lack of good management practices caused microbiological contamination of the aquatic environment.

## 1. Introduction

Feeding is considered of fundamental importance for all segments of animal production, including fish farming; it can represent more than 81% of production costs, depending on the adopted cultivation strategies [1]. Feeds formulated for fish must meet their nutritional needs; however, their composition can be compromised as it is possible to contain negative microbiological interference, possibly caused by inadequate storage [2]. Fungi and their mycotoxins can not only influence the texture and chemical composition of fish feeds, but they can also stimulate the proliferation of tumor cells in fish [3,4]. Likewise, some mycotoxins can be toxic to nervous, hepatic and renal system cells [5,6,7], in addition to reducing immunity [8].

Corn, wheat and soybean grains are commonly used as raw materials for formulating feeds and are more susceptible to fungal contamination at any stage of the fish farming production chain [9]. Corn is considered more susceptible to mycotoxin contamination when compared with rice [10]. Fish farms are favorable places for fungal growth, as they provide conditions of high temperature and humidity [6]. The quality of the feed will also depend on the sanitary control adopted with the raw material, from manufacture to grain damage during harvesting or handling [11]. When exposed to low-quality feeds and inputs, animals become susceptible to various diseases and toxic effects caused by fungi and their mycotoxins [12]. In Brazil, there are no specific maximum tolerated limits (MTLs) for fish, although to exemplify, there are MTLs for dairy products up to 10 µg kg^−1^ of Aflotoxins (B_1_, B_2_, G_1_ and G_2_); for grains up to 20 µg kg^−1^ of Ochratoxin A; and for cereals up to 750 µg kg^−1^ of Deoxynivalenol (DON) and up to 2.0 × 10^3^ µg kg^−1^ of Fumonisins (B_1_ + B_2_) [13].

Mycotoxins are secondary toxic metabolites produced by a variety of filamentous saprophytic fungi, especially those of the genera Aspergillus, Fusarium and Penicillium [14]. Due to the ecotoxicological risk they represent for human and animal health, there is a growing concern among importing countries regarding their presence in food, leading to the elaboration of legislation with the implementation of MTLs of mycotoxins in food. In Brazil, the MTLs for mycotoxins in food are defined by Resolution No. 7 of 18 February 2011, which defines admissible levels in products ready to be offered to the consumer and in raw materials [15]. However, there is no legislation for the presence of these metabolites in fish intended for human consumption.

One way to verify the ecotoxicological risk in aquatic ecosystems is to analyze anomalies in the blood of fish and amphibians, because they are considered excellent environmental bioindicators because they are highly sensitive to the quality of the environment, such as water quality [16,17]. The skin of these animals is thin and rich in blood vessels, and it is through it that amphibians interact with the environment. In the current study, the blood of tambaqui (*Colossoma macropomum*) was studied because it is the species raised in fish farms, and frogs of the species *Leptodactylus petersii* were studied since they are widely distributed in Amazonian streams and associated with the edges of fishponds [18].

Therefore, it is no use for the fish farmer to acquire quality feed even if they store it improperly. It is necessary to understand that the contamination of food inputs by mycotoxins can affect the health of fish, with implications for their productive efficiency, growth reduction, immunodeficiency, reduction of blood cells, lethargy, yellowish pigmentation related to jaundice and mortality [19].

Studies on the occurrence of mycotoxigenic fungi and mycotoxins in fish feed in Rondônia state are still insufficient, despite the fact that Rondônia has great potential for grain production and fish farming. Therefore, this study aimed to evaluate the occurrence of mycotoxigenic fungi in fish farm water and mycotoxins in feeds for farmed tambaqui (*Colossoma macropomum*).

## 2. Materials and Methods

### 2.1. Study Area

This study was conducted on fish farms in Rondônia state, Brazil, in the municipalities of Ji-Paraná, Presidente Médici, Urupá, Vale do Paraíso, Ouro Preto do Oeste, Teixeirópolis, Mirante da Serra, Urupá and Nova União. On average, the fish farms adopted a semi-intensive production system (maximum 0.6 kg m^−2^ per year with an annual cycle), covered up to 5 hectares of water surface, were distributed in semi-excavated ponds with an average depth of 1.60 m, and were intended to farm tambaqui (*Colossoma macropomum* Cuvier, 1818). In the visits to the properties, epidemiological data were collected regarding the type of creation, type of feeding, and storage conditions of the feed for farmed tambaqui (*C. macropomum*).

Concerning the climate of Rondônia state is classified in the Köppen system as a predominant type of Am—Tropical Rainy Climate [20]. However, the average climatological air temperature in the coldest month is greater than 18 °C (megathermal), and there is a well-defined dry season when there is a moderate water deficit with rainfall indices lower than 50 mm month^−1^ and between 1400 and 2600 mm year^−1^, while the average monthly air temperature ranges from 24 to 26 °C [21]. 

Rainfall data during the development of the current study were obtained by the Instituto Nacional de Pesquisas Espaciais (INPE) at the Estação de Previsão do Tempo e Estudos Climáticos (CPTEC) in Ouro Preto do Oeste city, Rondônia state, Brazil [20].

### 2.2. Quantification and Qualification of Free-Living Fungi in Fishponds

This study was developed in a completely randomized factorial design of 40 × 3 × 3 (40 fish farms, 3 fishponds and 3 replications per fishpond). A total of 40 fish farms were defined for data collection based on the availability of the Autarquia Empresa de Assistência Técnica e Extensão Rural (EMATER) of Rondônia state, and the fish farms are commercially active. Sample collections were conducted in the two Amazonian hydrological seasons: dry (May to August 2022) and rainy (September 2021 to April 2022). A total of 3 water samples were obtained from three different points in the fish farms. In other words, the freshwater collection points considered were the supply channel, the drainage pipe and the water column of the fishponds.

In this study, as suggested by Costa et al. [22], the fishponds are supplied in an interconnected way, in which the supply dam supplies freshwater to the first fishpond and, from then on, freshwater from one fishpond supplies the other. Then, the freshwater contained in the last fishpond passed through all the previous fishponds. Based on this context, sampling took place in alternate fishponds.

Concerning the composition of the qualitative samples, horizontal and vertical drags were carried out on the water surface. Each quantitative sample was obtained in a plankton net (50 μm mesh) with the aid of a graduated bucket. In this study, the count focused on free-living fungi. The samples were immediately stored in polyethylene terephthalate flasks, which were kept at 7 °C in cooler-type thermal boxes and sent to the laboratory.

It is important to inform readers that the free-living fungi were observed without the use of fixatives to avoid changes in their morphological characteristics. The analyses were performed with the aid of a trinocular stereoscopic microscope (Sigma, Japan) with 10× magnification and equipped with a digital camera. From there, photomicrographs of the images obtained were created using a professional still camera (Canon EOS Rebel T8i EF-S 18–55 mm). To contribute to the interpretation, the photomicrographs were analyzed using the Olympus Stream image analysis software.

Regarding the identification of free-living fungi, the water samples were concentrated by sedimentation, and the supernatant was discarded. Fungi were counted in Petri dishes and identified in triplicate in 1 mL Neubauer chambers under microscopic visualization; species identification was performed according to the taxonomic keys of Peach [23], Klich [24] and Milner [25], respectively. The free-living fungi count was shown at the taxonomic levels of species, family, order, class and phylum; in turn, the density was shown in colony-forming units per mL (CFU mL^−1^) in the different seasons (rainy and dry).

### 2.3. Ecotoxicological Risk Biomonitoring

Environmental biomonitoring of the ecotoxicological risk that mycotoxigenic fungi can cause to aquatic fauna was verified through micronucleus (MN) testing and other abnormalities associated with blood cells. Mutation counts were carried out in the peripheral blood of farmed fish and semi-aquatic frogs that lived along the edge of fishponds; the environmental conditions considered were above 1.0 and below CFU mL^−1^ in log_10_^(x+1),^ in addition to seasonal factors. In total, blood cells from 60 specimens of tambaqui *Colossoma macropomum* (Characiformes: Serrasalmidae) with 2.25 ± 0.25 kg and 130 specimens of *Leptodactylus petersii* (Anura: Leptodactylidae) with 32.0 ± 6.0 g were analyzed. For these procedures, this proposal was first submitted to the Chico Mendes Institute for Biodiversity Conservation (SisBio platform) and received approval under protocol No. 84820-1. This study was also submitted to the Ethics in Animal Use Committee of the Universidade Federal de Rondônia, Brazil. This proposal was approved under protocol No. 84820-1/UNIR/2021.

Immediately after capture, the animals were immobilized using their hands and nitrile gloves, and, in a tray moistened with water, 10 μL of peripheral blood was collected by puncturing the tail vein (at 45° on its ventral side). The blood was dripped directly onto the slides, and the smears were performed in duplicate for each animal. Immediately after blood collection, the animals were returned to their cages. The biological material was sent to the laboratory for a period of 12 h at room temperature. Then, the slides were hydrated for 5 min in distilled water and subsequently stained with the Quick Panotic LB kit. This kit is composed of three containers: Triarylmethane (0.1%), Xanthenes (0.1%) and Thiazines (0.1%). The slides were immersed 30 times in each container with submersion for one second in the sequence described above, according to Meneguetti et al. [26]. Subsequently, the slides were washed in deionized water at pH 7.0 and dried at room temperature, as described by Meneguetti et al. [26].

On each slide, 2000 erythrocytes per animal were counted, and the frequencies of erythrocytes with abnormalities were recorded. The frequencies of MN, cells in apoptosis, pyknosis, karyorrhexis, necrosis, nucleoplasmic bridge, binucleated cells—in mitosis, and cells in interphase at cell budding were all counted according to the methodology of Silva et al. [27]. These records were performed in a Trinocular Stereoscopic Microscope (Sigma, USA) through a 100X objective in oil immersion.

### 2.4. Fish Feeds and Storage Conditions

The pellets analyzed were to feed omnivorous fish, more specifically, farmed tambaqui (*C. macropomum*). Extruded commercial feed was supplied, containing 36% crude protein at a feeding rate of 1.0% in relation to body weight (Table 1). Handmade feed was also found, containing 28% crude protein at a feeding rate of 1.0% in relation to body weight. This handmade feed was made from fish silage and fermented corn. The feeds were supplied twice a day, from 8 a.m. to 6 p.m. It is important to point out that it is important to present information on the guaranteed levels of the feeds provided by fish farmers in order to demonstrate that the farms adopt a standardized diet.

**Figure 1:**
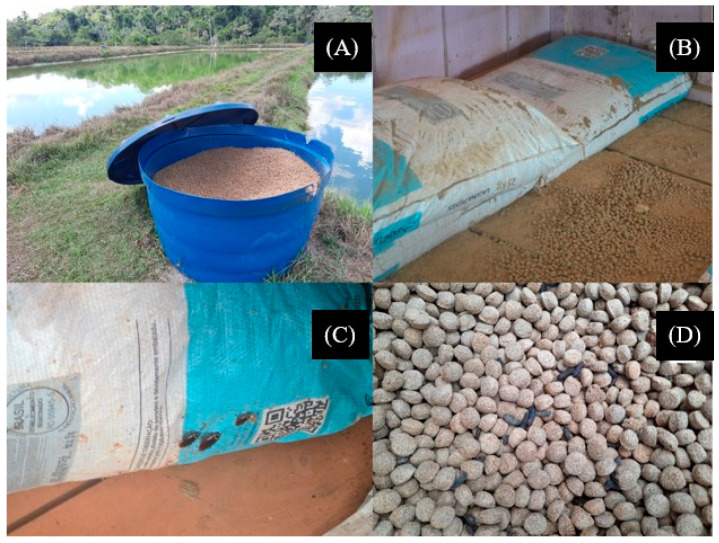
Feed storage conditions in fish farms from Rondônia state: feeds stored in polyethylene water tanks very close to the fishponds water (**A**); feed stored inside small covered rooms (**B**), although subject to insect contact (**C**); and also to humidity and high temperatures (**D**).

During visits to 40 fish farmers, it was possible to verify the storage conditions of the feed, and only one fish farm stored it properly. It was common to observe the storage of feed outside the bags; the feeds were stored in polyethylene water tanks very close to the fishpond’s water (Figure 1A). In some fish farms, feed bags were observed under the floor of the sheds without any protection against humidity (Figure 1B). Most of the time, the feed bags were stored inside small, covered rooms, although they were subject to contact with rats, cockroaches and woodworms, in addition to conditions of humidity and high temperatures (Figure 1C,D).

### 2.5. Determination of Mycotoxins in Fish Feeds: Acquired, Quantification and Chromatographic Conditions

Those feeds for farmed tambaqui (*C. macropomum*) confirmed with filamentous fungi were sent to the Mycotoxicological Analysis Laboratory at the Universidade Federal de Santa Maria (UFSM) in Santa Maria, Brazi, for extraction, detection and quantification of mycotoxins.

The pellets of fungal feeds were submitted to liquid chromatography coupled to mass spectrometry (LC–MS/MS) by the POP 40 technique (Rev. 09) to quantify AFB_1_ (1 μg kg^−1^ per 94.5%), AFB_2_ (1 μg kg^−1^ per 80%), and AFG_1_ (1 μg kg^−1^ per 88.5%); by the POP 42 technique (Rev. 07) to quantify 200 μg kg^−1^ per 80% of the total; by the POP 45 technique (Rev. 06) to quantify FB_1_ (125 μg kg^−1^ per 90.8%), FB_2_ (125 μg kg^−1^ per 89.8%); finally, by the POP 44 technique (Rev. 09) to quantify 2.0 μg kg^−1^ per 80% of the total, in relation to the limit of quantification (LQ)/coefficient of recovery (CR), respectively. 

For the quantification of OTA, chromatographic elution was performed on a Kromasil C18 column at 40 °C with a flow rate of 0.8 mL min^−1^ with a standardized fluorescence detector with excitation and emission wavelengths of 333 and 460 nm, respectively [18]. The limit of detection (LOD) was determined by standard injections of AFB_1_ and OTA solutions in decreasing order of concentration until the peak signal of the analyte reached a height three times greater than the baseline signal in the retention time of the compound of interest.

The methods of extraction, detection and quantification of Deoxynivalenol (DON), Fumonisin (FB) in LC–MS/MS, were adopted from Tardieu et al. [28], respectively, and adapted for extruded and pelleted feeds.

### 2.6. Database and Statistical Analysis

The data obtained were stored and organized in the Epi info^TM^ software, version 3.5.3—2011 (OS: MS-Windows, C Sharp programming language).

To determine the variation of free-living fungi in fishponds, the counts were conducted twice, in different hydrological seasons (rainy and dry), and the identifications were carried out by the same individual observer. Richness was demonstrated in terms of average counts and densities for each location, with the standard deviation between the two counts (rainy and dry). Regarding the averages of the different seasons, they were compared by Student’s *t*-test, with differences considered statistically significant for *p* < 0.05.

Qualitative and quantification analyses of mycotoxins in fish feed were conducted in triplicate, with the results shown as average (μ) and standard deviation ± SD (σ). Concerning mycotoxin statistics, averages were analyzed by one-way ANOVA. The homoscedasticity and normality of the residues were evaluated by Bartlett and Shapiro–Wilk tests (α = 0.05), respectively. Pearson’s correlation was applied to verify possible correlations between the mycotoxins found and the feeds.

All statistical analyses were performed using RStudio Development Core Team, version 3.5.3.

## 3. Results

### 3.1. Climatic Conditions

**Figure 2:**
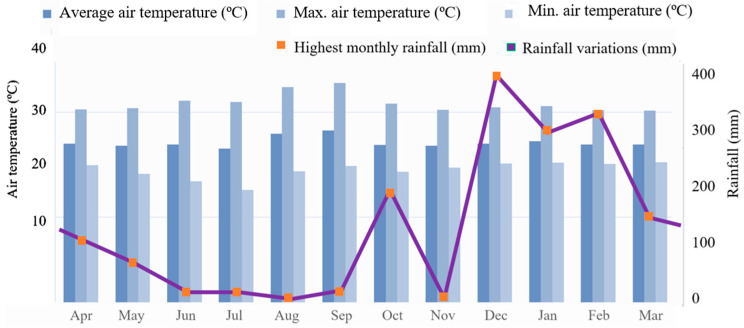
Monthly averages of rainfall (mm) and air temperature in the interior of Rondônia state in the different hydrological seasons (rainy and dry) in the years 2021 and 2022.

The average air temperature, during the coldest month, exceeded 18 °C (megathermal), and a well-defined dry season occurs when there is a moderate water deficit with rainfall indices below 50 mm month^−1^. The average annual rainfall varied between 1400 and 2600 mm year^−1^, while the monthly average air temperature varied between 24 and 26 °C (Figure 2). Regarding meteorological data, the months of October 2021 (200 mm), December 2021 (189.50 mm), January 2022 (336.05 mm) and February 2022 (359.66 mm), showed the highest precipitation averages. While the months of August and September 2021 showed the highest average temperatures, >35 °C, respectively. There were no significant variations in the other months (Figure 2).

### 3.2. Free-Living Fungi in Freshwater Fishponds

**Figure 3:**
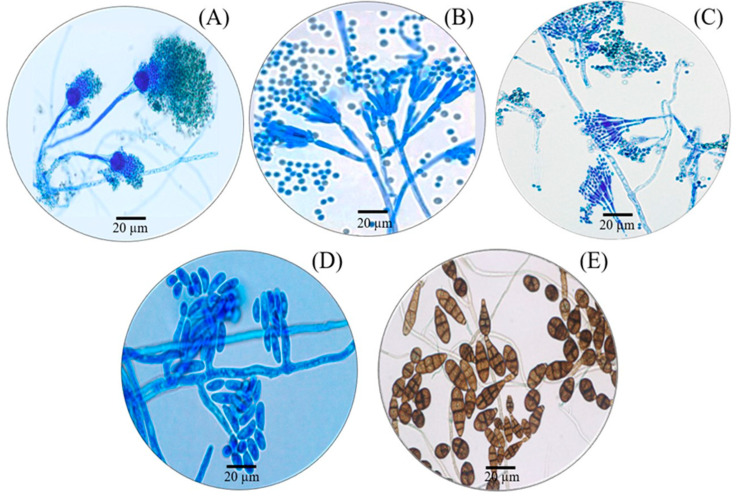
Photomicrographs of free-living fungi: *Aspergillus fumigatus* (**A**), *Penicillium citrinum* (**B**), *Penicillium implicatum* (**C**), *Fusarium oxysporum* (**D**) and *Alternaria alternata* (**E**).

Water samples were collected from forty fish farms, and fungi species were found in the freshwater from eight fish farms. A total of five species of free-living fungi were found, which belong to three families, two orders, four classes and one phylum. The species found were *Aspergillus fumigatus*, *Penicillium citrinum*, *Penicillium implicatum*, *Fusarium oxysporum* and *Alternaria alternata* (Table 2; Figure 3).

The fungi species were counted in water samples at 35.14 CFU mL^−1^ in the rainy season and 24.69 CFU mL^−1^ in the dry season (Table 3). *A. fumigatus* was the species with the highest count (12.81 CFU mL^−1^ in the rainy season and 7.47 CFU mL^−1^ in the dry season) and a greater predominance in the rainy season (like other fungi species), followed by *F. oxysporum* (9.07 CFU mL^−1^ in the rainy season and 5.77 CFU mL^−1^ in the dry season), *A. alternata* (8.54 CFU mL^−1^ in the rainy season and 5.97 CFU mL^−1^ in the dry season), *P. citrinum* (2.97 CFU mL^−1^ in the rainy season and 3.12 CFU mL^−1^ in the dry season), and *P. implicatum* (1.74 CFU mL^−1^ in the rainy season and 2.36 CFU mL^−1^ in the dry season).

**Figure 4:**
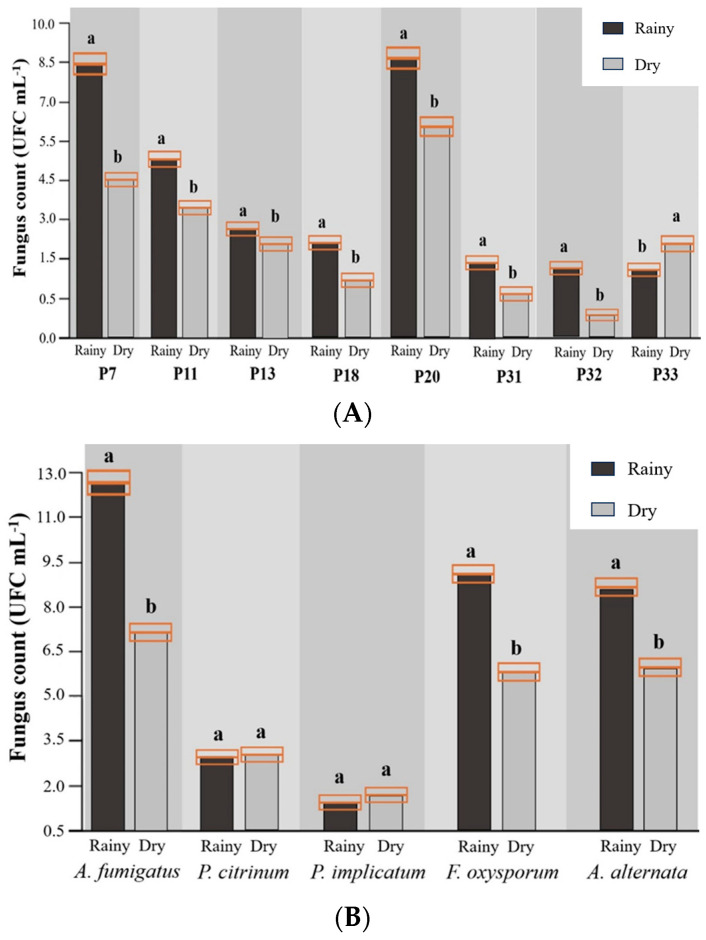
Abundance of free-living fungi per fish farm freshwater (**A**) and per species of fungi (**B**) in rainy and dry seasons. Subtitle: If there are different letters at the top of the fungus count columns, there is a statistical difference according to the Student’s *t*-test (*p* < 0.05).

In all fish farms, there was a higher abundance of fungi species in the rainy season (Figure 4A), while in terms of abundance by species, with the exception of *P. citrinum* and *P. implicatum* (*p* > 0.05), the other species had higher abundances in the rainy season (Figure 3B). It was observed that the more predominant species, *A. fumigatus* and *F. oxysporum,* had higher abundances in the rainy season, while the two less prevalent species, *P. citrinum* and *P. implicatum*, showed no difference (*p* > 0.05) between the rainy and dry seasons.

### 3.3. Ecotoxicological Risk

**Figure 5:**
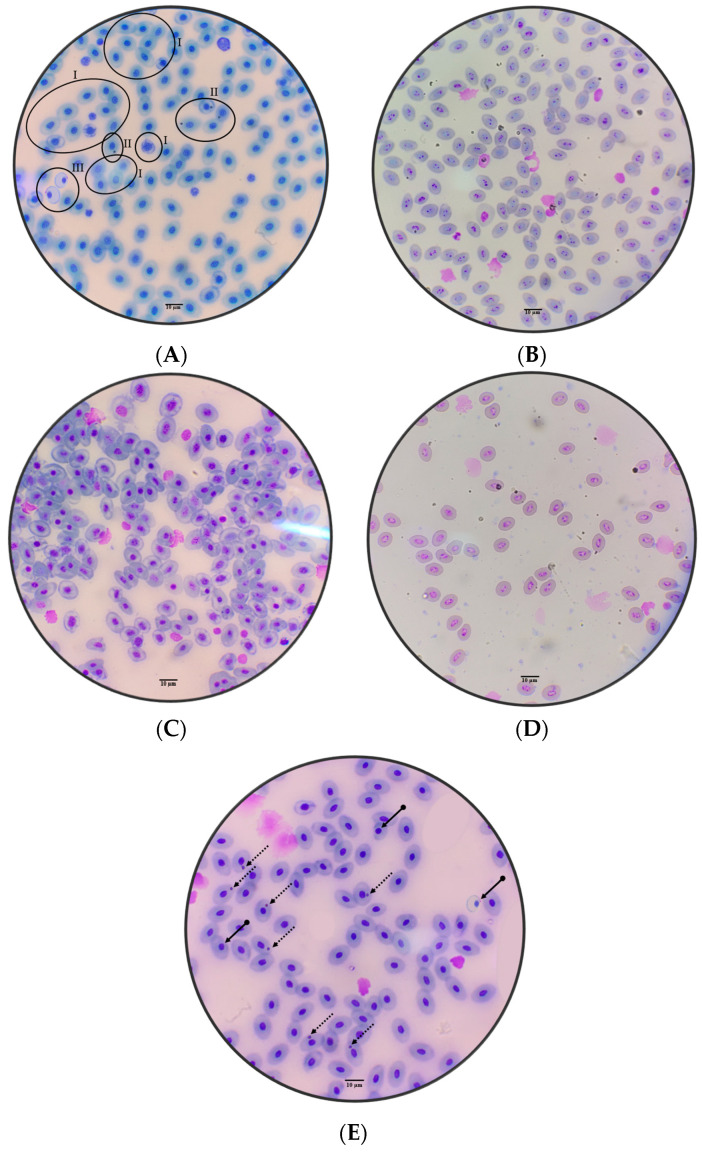
Photomicrographs of abnormalities observed in tambaqui (*Colossoma macropomum*) blood cells: apoptosis I, micronucleated erythrocytes II and pyknosis III (**A**), and impressiving image of almost every cell undergoing karyorrhexis (**B**), and photomicrographs of abnormalities observed in *Leptodactylus petersii* blood cells: impressiving image of almost every cell undergoing apoptosis (**C**) and karyorrhexis (**D**); dotted arrows point to micronucleated erythrocytes; and arrows with rounded ends point to pyknosis (**E**).

On ecotoxicological risk analysis, abnormal blood cell counts are signs of mutagenicity; abnormalities were found in apoptosis, micronucleated erythrocytes, pyknosis and karyorrhexis in the blood cells of tambaqui (*C. macropomum*) and *Leptodactylus petersii* (Figure 5A–E).

**Figure 6:**
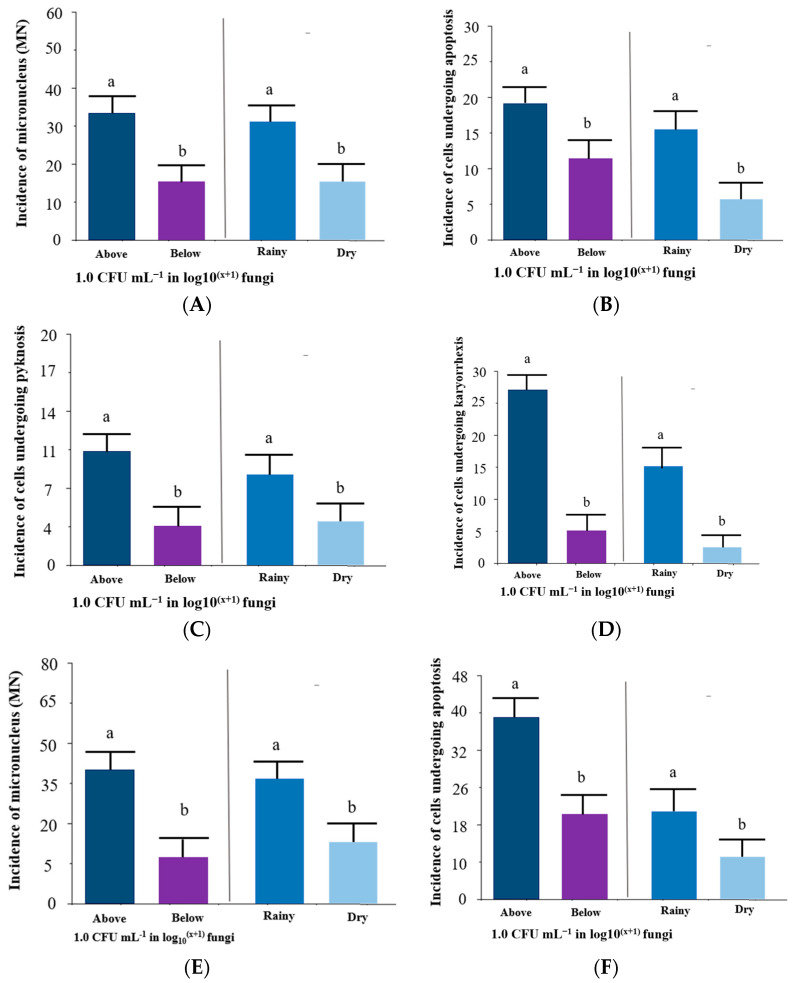

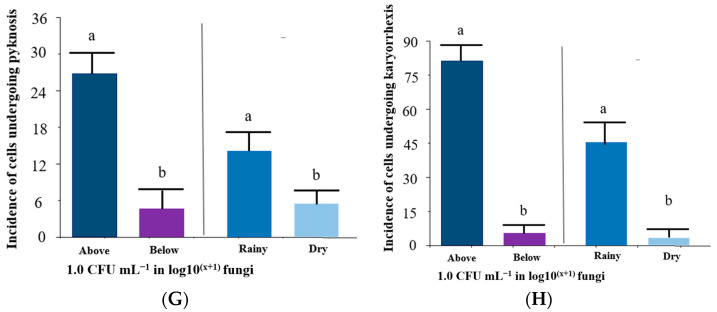
Incidences of abnormality in tambaqui (*Colossoma macropomum*) blood cells, micronucleus (MN) (**A**), cells undergoing apoptosis (**B**), pyknosis (**C**), and karyorrhexis (**D**); and incidences of abnormality in *Leptodactylus petersii* blood cells, micronucleus (MN) (**E**) cells undergoing apoptosis (**F**), pyknosis (**G**) and karyorrhexis (**H**). Subtitle: Statistical comparison, Student’s *t*-test, *p* < 0.05.

Figure 6A–D shows the incidence of abnormalities in tambaqui (*C. macropomum*) blood cells. The incidence of micronucleus (MN) was higher (32 cells per slide in the condition above 1.0 CFU in log_10_^(x+1)^ fungi) compared to below 1.0 CFU in log_10_^(x+1)^ fungi, as well as being higher (30 abnormal cells per slide) in the dry season (*p* < 0.05) (Figure 6A). Regarding the incidence of cells undergoing apoptosis, it was higher (19 cells per slide in the condition above 1.0 CFU in log_10_^(x+1)^ fungi) compared to below 1.0 CFU in log_10_^(x+1)^ fungi, as well as being higher (15 abnormal cells per slide) in the dry season (*p* < 0.05) (Figure 6B). The incidence of cells undergoing pyknosis was higher (eleven cells per slide in the condition above 1.0 CFU in log_10_^(x+1)^ fungi) compared to below 1.0 CFU in log_10_^(x+1)^ fungi, as well as being higher (nine abnormal cells per slide) in the dry season (*p* < 0.05) (Figure 6C). Concerning the incidence of cells undergoing karyorrhexis (27 cells per slide in the condition above 1.0 CFU in log_10_^(x+1)^ fungi) compared to below 1.0 CFU in log_10_^(x+1)^ fungi, as well as being higher (16 abnormal cells per slide) in the dry season (*p* < 0.05) (Figure 6D).

### 3.4. Mycotoxins in Fish Feeds

A total of forty fish farms were visited, although only twenty-one of them provided commercial feed. Another five fish farms elaborated on handmade feeds, while the remaining fourteen did not offer fish feeds and only fermented corn once a day. A total of 16 feed samples were collected, with 500 g obtained from each feed bag. 

The isolated samples of fish feed with identified filamentous fungi were sent to the Mycotoxicological Analysis Laboratory at the Universidade Federal de Santa Maria (UFSM), located in Santa Maria, Brazil. In this laboratory, the qualification and quantification of mycotoxins in feeds for farmed tambaqui were conducted. The occurrence of mycotoxicological contamination was confirmed in 81.25% of the analyzed samples. The quantified mycotoxin was Fumonisins B_1_ + B_2_ (375 to 1418 μg kg^−1^) (Table 4).

**Figure 7:**
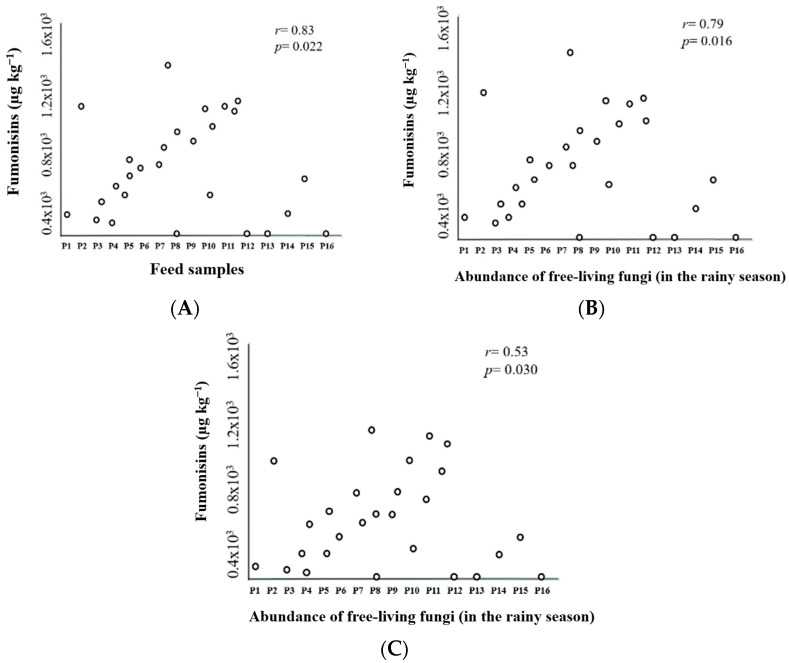
Pearson’s correlation analyzes the relationship between fish farm feed samples and Fumonisins (**A**), the abundance of free-living fungi in the rainy season and Fumonisins (**B**), and this same correlation in the dry season (**C**).

Pearson’s correlation analysis showed a significant correlation between fish farm feed samples and Fumonisins (r = 0.83; *p* = 0.022). There were also significant correlations between the abundance of fungi in fishpond water and Fumonisins (r = 0.79; *p* = 0.016 in the rainy season and r = 0.53; *p* = 0.030 in the dry season) (Figure 7A–C).

## 4. Discussion

The biological quality of the freshwater is a constant concern in fish farming because, when it is of poor quality, there may be decreases in the productive performance and mortality of the fish. Some factors cause a reduction in production and profitability [29]. Therefore, surface water pollution is an environmental problem worldwide because fish farm ponds represent artificial environments with a constant high concentration of organic matter. In addition, these cultivation environments receive significant volumes of industrial, agricultural and domestic effluents [30]. Most aquatic organisms can respond directly to changes in the physical, chemical and biological profile of freshwater [31]. Among the aquatic organisms used as bioindicators, free-living toxigenic filamentous fungi have been highlighted in recent decades [32,33]. These organisms are part of the protozooplankton in fishpond water.

Regarding the implications of free-living fungi found in fish farm water, *Aspergillus fumigatus* is one of the most common species to cause disease in individuals with immunodeficiency [34]. *Penicillium citrinum* is a species of anamorphic and mesophilic fungus of the genus Penicillium that produces Tanzawaic acid AD, ACC, Mevastatin, Quinocytrinin A, Quinocytrinin B and nephrotoxic citrinin. *P. citrinum* is often found in moldy citrus fruits and occasionally occurs in spices and cereals used in commercial feed manufacturing. This species also causes mortality in aquatic invertebrates. Due to its mesophilic character, *P. citrinum* occurs worldwide [35].

The species *Penicillium implicatum* is a fungus of the genus Penicillium that produces citrina, which is why it causes the rotting of organic matter at the bottom of fishponds [36]. While *Fusarium oxysporum* is a species of ascomycete fungus, it comprises all species, varieties and forms recognized by Wollenweber and Reinking within an infrageneric grouping called Section Elegans. It is part of the Nectriaceae family [37]. And finally, the species *Alternaria alternata* is a fungus that causes leaf spots and other diseases in more than 380 species of host plants, including aquatic phanerogams. It is an opportunistic pathogen in numerous hosts, causing whitish spots and decay of organic matter in fishponds [38].

As far as the ecotoxicological risk analysis is concerned, the current study revealed mutations in the blood cells of farmed fish and semi-aquatic frogs, *Leptodactylus petersii.* Therefore, it is indicative of toxicity due to some pollution event, and the focus was given to the presence of mycotoxigenic fungi in poorly stored food given to fish grown in the ponds, such as the farmed tambaqui (*C. macropomum*). So, in addition to the financial loss, the fish farmers were contaminating the water with fungal feed, and the species of fungi found are capable of synthesizing very harmful toxins in the water. Amphibians are considered excellent bioindicators of environmental conditions, as they are highly sensitive to sudden changes and pollution. Regarding the frog, *L. petersii* is perhaps one of the most sensitive amphibians because its habit is semi-aquatic, that is, it is sensitive to aquatic and terrestrial environments [18,27]. As for the tambaqui (*C. macropomum*), in addition to being subjected to confinement, the cultivation ponds are built in the lower regions of the relief so that there is no lack of dammed water, therefore it receives various polluting loads from agriculture and domestic and industrial effluents by rain leaching. Another important factor of the tambaqui (*C. macropomum*) is that it is a filter fish that retains more particles suspended in the water, which enhances its sensitivity to pollutants [16].

The continuous exposure of aquatic animals to water contaminated with mycotoxigenic fungi may be triggering previously unknown diseases and high mortality. Mycotoxins can bioaccumulate in tissues, causing cellular damage. inhibiting the action of repairing enzymes, modifying DNA and negatively interfering in the physiology of exposed animals [39]. The normal amount of micronucleus found in erythrocytes varies according to species and is related to the repair capacity of the cell. Exposure to xenobiotics affects this repair capacity; therefore, erythrocytes that have undergone mutation will show abnormal differences under different conditions of pollution or seasonality. For example, micronucleated erythrocytes are a reflection of the genotoxic effects to which the organism is exposed [40].

Li et al. [41] studied the adverse effects of cyclophosphamide (a carcinogenic drug) in different doses on *Danio rerio*. The fish were banned days after the beginning of the experimental tests with histopathological changes in the retina and liver, although first a significant increase in abnormal cells in the blood, such as micronucleus, apoptosis, pyknosis and karyorrhexis, including malformations and altered transcriptomes, was documented. Therefore, we recommend that the ecotoxicity of mycotoxigenic fungi be tested in the laboratory and then continuous environmental biomonitoring be conducted in fish farms.

According to AnvariFar et al. [42], cells in apoptosis (cell death), pyknosis and karyorrhexis are indicative of self-destruction of cells that aim to maintain the homeostasis of the organism and occur in a programmed manner. These three mutations showed a higher incidence in conditions with mycotoxigenic fungi and in the rainy season. Therefore, there was a stimulus for pathological processes in the organism. Karyorrhexis is a type of cell mutation that destructively fragments the erythrocyte nucleus; its chromatin is irregularly distributed throughout the cytoplasm, causing cell death. It is usually preceded by pyknosis and followed by karyolysis and can occur as a result of programmed cell death, senescence, or necrosis [43].

The main mycotoxigenic fungi belong to the genera Aspergillus, Fusarium and Penicillium in fish farming and are considered bioindicators of contaminants in inputs such as feed or even their surplus of organic matter. Fungi are organisms without chlorophyll and can be unicellular or multicellular. They are free-living or not, and they are found in the most varied environments, especially in humid places rich in organic matter [32]. Concerning the mycotoxins that can be synthesized, Aspergillus (Aflatoxins), Fusarium (Fumonisins, Trichothecenes and Zearalenone), and Penicillium (Ochratoxin A). These mycotoxins have carcinogenic, teratogenic and mutagenic properties [5,6,7].

As found in the current study, the mycotoxin contamination levels in fish feeds did not exceed the maximum limits established by Brazilian legislation, as well as in the study by Nogueira et al. [11], in which commercial fish farms in Rondônia and Rio Grande do Sul states (Brazil) were analyzed. Ordinance MA/SNAD/SFA No. 07 of November 9, 1988 of the Ministério da Agricultura, Pecuária e Abastecimento from Brazil [44] establishes only the maximum levels for AFB_1_ (20 µg kg^−1^) in feed intended for animal consumption, and there is no guidance for other mycotoxins. However, concerning the maximum limits established (MTL) by the European Commission through Recommendation No. 2006/576, the contamination levels for AFB_1_ and OTA are 65 µg kg^−1^ and 532% above the levels allowed for animal feed, respectively. The determination of maximum limits for OTA is fundamental for the fisheries sector. The European Commission No. 576 of 17 August 2006 establishes MTLs for mycotoxins produced by the genus *Fusarium* sp., for Fumonisins (B_1_ + B_2_) in products for feeding pigs, horses (equines), rabbits and companion animals (5000 µg kg^−1^), fish (10,000 µg kg^−1^), poultry, calves, lambs and kids (20,000 µg kg^−1^), and adult ruminants (50,000 µg kg^−1^) [3,4].

Pietsch et al. [45], when conducting a study of dietary trials to evaluate the effects of Zearalenone (ZEN) on *Cyprinus carpio*, found that concentrations similar to those found in the studied diets (332 µg kg^−1^) can cause adverse effects on the immune system in a short period of time. Likewise, when evaluating the effect of OTA at different concentrations (80 and 160 µg kg^−1^) for *Oreochromis niloticus* fingerlings, OTA can affect zootechnical parameters and gonadosomatic indices, as well as reduce fish feed conversion [46]. Deng et al. [47] performed dietary tests with different concentrations (19, 85, 245, 638, 793 and 1641 µg kg^−1^) of AFB_1_ for *Oreochromis niloticus*. The authors found that after the 20th week of supplementation, the fish showed reduced growth in addition to eating disorders, which caused a decrease in cytochrome P450 activity.

Research conducted in Brazil on the presence of AFB_1_ in fish feed commercialized in the Northeast region showed average levels of 3.8 µg kg^−1^ [48]. Barbosa et al. [49] found that 55 and 3.3% of feed samples marketed in the Midwest region were contaminated with AFB_1_ and OTA, respectively, at detectable levels. Nogueira et al. [11] studied commercial feeds from Rondônia and Rio Grande do Sul states (Brazil), and the results showed that 93.3% of the samples were contaminated at maximum levels of 16.5, 31.6 and 322 μg kg^−1^ for mycotoxins AFB_1_, OTA and ZEN, respectively. The same study showed that the younger the fish, the more they consumed high levels of AFB_1_, OTA and ZEN, causing serious ecotoxicological risk. In several regions of the world, studies confirm the presence of mycotoxins in fish feed, such as AFB_1_ (1.8 to 39.7 µg kg^−1^) in Kenya [50], ZEN (81.8 µg kg^−1^) in Poland [51], Fumonisin B_1_ (900.9 µg kg^−1^), Fumonisin B_2_ (220.6 µg kg^−1^), AFB_1_ (103.0 µg kg^−1^) and ZEN (4.5 µg kg^−1^) in Nigeria [52]. Therefore, compared to the current study, Fumonisins present minimum and maximum quantifications, respectively, of 375 µg kg^−1^ (P4) and 1418 µg kg^−1^ (P7). These are produced by fungi of the genus *Fusarium* sp. and have different structures related to A (A1–A4), B (B1–B4), C (C1–C4) and P10 [53]. These mycotoxins contribute to a series of consequences at the cellular level, such as the induction of apoptosis and carcinogenic effects [53].

The contamination of inputs and food with Fumonisins has been associated with several diseases that can affect the health of animals and humans. They have hepatotoxic and nephrotoxic effects in most animal species tested [54,55]. For example, in horses, they lead to the appearance of leukoencephalomalacia (ELEM); in pigs, they cause pulmonary edema (PPE). In sheep, rats and rabbits, they lead to renal toxicity and are also hepatotoxic [28]. They have also been epidemiologically related to human carcinogens. Fumonisins are cytotoxic and inhibit protein and DNA synthesis, promote oxidative stress, induce DNA fragmentation and interrupt the cell cycle [56]. They are analogs of sphinganine, inhibiting the biosynthesis of sphingolipids [53].

Continuing in the quantification context of Fumonisins in fish feeds, in an assay developed in the northern region of Croatia, the sensitivity of juvenile specimens of *Cyprinus carpio* to diets containing different concentrations of FB_1_ (0.5, 5.0, 10.0 and 100.0 mg kg^−1^) was measured. Although no deaths were observed, there was a decrease in weight gain, higher rates of opportunistic infections, hematological alterations and neurotoxicity, and such clinical signs were proportional to the ingested dose [57,58]. Fumonisin B_1_ had a carcinogenic effect on *Oncorhynchus mykiss* in a study conducted in the USA when associated with aflatoxin B_1_ and n-methyl-n’-nitro-nitrosoguanidine [59].

In a study carried out in Londrina, Brazil, Hashimoto et al. [60] observed that the co-occurrence of aflatoxin and Fumonisin in extruded and pelleted fish feeds may present a risk of toxic synergism even at levels lower than the limit allowed by Brazilian legislation. In Rio Grande do Sul state, the performance of silver catfish fingerlings treated with different concentrations of Fumonisins (20, 30 and 40 mg kg^−1^) was tested, confirming that diets containing 40 mg kg^−1^, despite not causing any deaths, led to changes in the parameters of final weight, weight gain and biomass, especially when in the juvenile stage [61].

The incidence of fumonisins in fish feed is related to the tendency to replace animal protein sources with corn, an important source of starch [62], this ingredient is essential for the expansion, agglutination and buoyancy of fish feed pellets [63]. It is important to inform that the minimum amount of starch to guarantee pellet buoyancy of at least 18 minutes is 20% [64]. Therefore, the factors nutritional needs, growth stage and eating habits of farmed fish determine which starch inclusion levels complement ingredients of animal origin [65].

In view of this, in most feed mills, corn is the main source of starch. Among the sources of starch, corn has the highest degree of contamination by different mycotoxins [12]. The presence of these mycotoxins in foods adopted in fish farming can be attributed to their inadequate storage temperature, and even at high temperatures in the industrial process of producing feed, some mycotoxins persist. The occurrence of these persistent metabolites may be conditioned by long storage times and interactions between microorganisms, although mainly between temperature and humidity [66]. This factor is aggravated by climatic conditions in the Amazon, such as the high rainfall in the rainy season, so information on the distribution of mycotoxins in agricultural products and commodities is essential [67]. Thus, there is a need for research to establish maximum limits of mycotoxins for fish farming, contributing to the feed quality provided. As well as the nutritional security of farmed fish.

Fish feed contaminated by mycotoxigenic fungi, a way to introduce pathogens and toxins to a fishpond, can accelerate the eutrophication process, thereby affecting water quality [68]. The color of the water can become cloudy with lower levels of dissolved oxygen, which can cause the death of the fish at any time; however, there is a greater risk at dawn, the most critical period of biochemical oxygen demand [69].

The surplus feed being allocated in the fishpond sediments may increase the levels of nitrogen and phosphorus, which may cause a series of problems, exemplified by the dangers of the increase in ammonia content and the proliferation of microalgae. Ammonia is a very restrictive toxicant for the life of fish, and many species do not support concentrations above 5 mg L^−1^, and values above 0.01 mg L^−1^ can be toxic to fish. However, total ammonia is divided into ionized ammonium (NH_4_) and non-ionized NH_3_, the latter having a toxic effect. Above pH 9, with each increase in Log_10_, the toxic potential increases 10× [70].

As for the proliferation of microalgae, eutrophication is a process of excessive multiplication of microalgae, common in aquatic ecosystems without as much movement as fishponds with low water renewal [71]. Although it means a large amount of organic matter is present in water, it can cause various harm to humans and nature itself. Health problems caused by cyanobacteria blooms and their cyanotoxins vary depending on the type of exposure, amount and type of toxin present [72]. Contact with cyanotoxins can cause death and illness; for example, their consumption can cause gastrointestinal symptoms, eczema, allergic irritations and severe cases of hepatotoxicity [73].

Another factor worth mentioning is that if the fish are not able to ingest the feed or are contaminated, they are discarded by the handlers. There is certainly a critical economic loss to fish farming. In a fish farm in Rondônia state, the cost of feeding the fish represents 81% of the production cost [1]. Confirming once again the need to make fish farmers aware of the commitment to proper storage of feed, which they sometimes deem to be expensive due to the rise in prices of inputs such as corn and soybeans. Therefore, a balanced diet with high nutritional standards is of no use, although if there is no adequate storage, it will not satisfy the needs of the fish. On the contrary, it may facilitate the entry of pathogens into the fishponds or cause an ecotoxicological risk, thus becoming a public and environmental health problem [74].

Therefore, it is suggested that further studies be conducted on the development of the occurrence of Fumonisins in the feed and muscle tissue of fish, as there is a lack of research on the residual effects, as well as an anatomopathological study to describe the lesions found after exposure to the toxic agent. Furthermore, little has been understood about the interactions and potential synergism of this mycotoxin with other substances.

## 5. Conclusions

Based on the results obtained, it can be concluded that free-living fungi can be used as bioindicators of water quality in fish farms. 

There was a correlation between the feed contaminated with Fumonisins and the abundance of free-living fungi, regardless of the hydrological season, despite higher values in the rainy season, which is also related to subjecting the feed to humidity and high temperatures. 

Concerning mutations in blood cells, in tambaqui (*C. macropomum*), a total of 159 anomalies were found, and in *Leptodactylus petersii,* 299 anomalies were found, with higher incidences in conditions above 1.0 CFU mL^−1^ in log_10_^(x+1)^ fungi and in the rainy season. Therefore, it is indicative of toxicity due to some pollution event, and the focus was given to the presence of mycotoxigenic fungi in poorly stored food given to fish grown in the ponds. So, in addition to the financial loss, the fish farmers were contaminating the water with fungal feed, and the species of fungi found are capable of synthesizing very harmful toxins in the water. 

Consequently, in addition to the loss of feed quality, they caused severe contamination of pond water by Fumonisins. Therefore, the lack of good management practices caused microbiological contamination of the aquatic environment.

Although the levels of contamination by Fumonisins found did not exceed the maximum tolerable limits established by Brazilian legislation, they exceeded the detection limit legislation of the European Commission and the USA. This fact is an impediment to fish exports from Rondônia state. 

Therefore, further studies are needed on the adverse effects of Fumonisins on the productivity and performance of fish, as well as the establishment of minimum doses capable of causing hepatotoxicity, nephrotoxicity, carcinogenesis and bioaccumulation. Therefore, strict control of the feed quality used in fish farming is necessary in order to prevent contamination by mycotoxigenic fungi, mitigate risks to the health of fish and humans (fish consumers), and improve water quality.

## Figures and Tables

**Table 1:** Guarantee levels of the commercial feed for farmed tambaqui (*Colossoma macropomum*).

Feed Composition	Content (g kg^−1^)	Feed Composition	Content (g kg^−1^)
Dry matter (g)	910.0	Ethereal extract (min, g)	80.0
Crude protein (min, g)	360.0	Calcium (max, g)	35.0
Fibrous matter (max, g)	95.0	Calcium (min, g)	20.0
Mineral matter (max, g) ^1^	15.0	Phosphorus (min, g)	15.0

^1^ Amount of nutrients per kg for the crude protein ration (36%). Pantothenic acid (min)—3.00 mg; Biotin (min)—50 mg; Choline (min)—290 mg; Vitamin A (min)—28,000 IU; Vitamin B_1_ (min)—2.00 mg; Vitamin B_12_ (min)—4.00 mg; Vitamin B_2_ (min)—3.00 mg; Vitamin B_6_ (min)—2.00 mg; Vitamin D_3_ (min)—5000 IU; Vitamin E (min)—45.00 IU; Vitamin K_3_ (min)—2.00 mg; Vitamin C (min)—500 mg; Copper (min)—10.00 mg; Total iron (min)—90 mg; Iodine (min)—0.40 mg; Niacin (min)—50.00 mg; Manganese (min)—10.00 mg; Zinc (min)—180 mg; Selenium (min)—0.60 mg. Source: Fish processing units in the Vale do Paraíso and Ariquemes, RO, Brazil.

**Table 2:** Taxa of free-living fungi identified in fish farm freshwater.

Species	Taxonomic Classification			
	Family	Order	Class	Phylum
*Aspergillus fumigatus* Fresen. (P. Micheli, 1729)	Trichocomaceae	Eurotiales	Ascomycetes	Ascomycota
*Penicillium citrinum* (Thom, C. 1910)	Trichocomaceae	Eurotiales	Eurotiomycetes	Ascomycota
*Penicillium implicatum* (Biourge, P. 1923)	Trichocomaceae	Eurotiales	Eurotiomycetes	Ascomycota
*Fusarium oxysporum Schlecht. emend. Snyder & Hansen*	Nectriaceae	Hypocreales	Sordariomycetes	Ascomycota
*Alternaria alternata* (Fr.:Fr.) Keissl.	Pleosporaceae	Pleosporales	Dothideomycetes	Ascomycota

**Table 3:** Counting in the rainy and dry seasons of free-living fungi in fish farm freshwater.

Count (UFC mL^−1^ in log_10_^(x+1)^)		Species				
Fish Fams	Seasons	*A. fumigatus*	*P. citrinum*	*P. implicatum*	*F. oxysporum*	*A. alternata*
P7	Rainy	3.22	0.44	0.10	2.95	1.63
	Dry	1.00	0.20	0.15	1.00	2.20
P11	Rainy	2.17	0.26	0.11	0.80	1.55
	Dry	1.33	0.22	0.30	1.11	0.34
P13	Rainy	0.12	0.00	0.00	1.55	0.96
	Dry	0.00	0.20	0.00	1.10	0.90
P18	Rainy	2.10	0.60	0.43	2.00	1.50
	Dry	1.16	0.50	0.77	2.25	1.00
P20	Rainy	3.22	1.33	0.81	1.77	1.43
	Dry	3.07	1.00	0.92	0.00	1.04
P31	Rainy	0.85	0.00	0.00	0.00	0.55
	Dry	0.40	0.10	0.00	0.10	0.24
P32	Rainy	0.33	0.20	0.15	0.00	0.67
	Dry	0.10	0.00	0.00	0.10	0.12
P33	Rainy	0.80	0.14	0.14	0.00	0.25
	Dry	0.41	0.90	0.22	0.11	0.13

**Table 4:** Quantification and limit established for mycotoxins in feeds for farmed tambaqui (*Colossoma macropomum*).

Fish Farms	Mycotoxins (μg kg^−1^)					
	AFB_1_	AFB_2_	AFG_1_	FB (B_1_ + B_2_)	DON	OTA
P1	<LQ	<LQ	<LQ	463 ± 70	<LQ	<LQ
P2	<LQ	<LQ	<LQ	1215 ± 184	<LQ	<LQ
P3	<LQ	<LQ	<LQ	430 ± 65	<LQ	<LQ
P4	<LQ	<LQ	<LQ	375 ± 57	<LQ	<LQ
P5	<LQ	<LQ	<LQ	922 ± 140	<LQ	<LQ
P6	<LQ	<LQ	<LQ	852 ± 129	<LQ	<LQ
P7	<LQ	<LQ	<LQ	1418 ± 215	<LQ	<LQ
P8	<LQ	<LQ	<LQ	<LQ	<LQ	<LQ
P9	<LQ	<LQ	<LQ	920 ± 139	<LQ	<LQ
P10	<LQ	<LQ	<LQ	517 ± 78	<LQ	<LQ
P11	<LQ	<LQ	<LQ	1262 ± 185	<LQ	<LQ
P12	AI	AI	AI	0	AI	AI
P13	AI	AI	AI	0	AI	AI
P14	<LQ	<LQ	<LQ	474 ± 72	<LQ	<LQ
P15	<LQ	<LQ	<LQ	909 ± 138	<LQ	<LQ
P16	<LQ	<LQ	<LQ	1378 ± 209	<LQ	<LQ

Subtitle: Aflatoxins (AFB_1_, AFB_2_, AFG_1_ and AFG_2_), DON—Deoxynivalenol, FB—Fumonisins, OTA—Ochratoxin A, <LQ—lowest concentration of compound per substance that can be determined with an acceptable level of accuracy and accuracy.

## Data Availability

Data will be made available on request.
